# Tyrosine kinase inhibitors in Kosaki/Penttinen syndromes: new reports, follow-up of treated individuals and literature review

**DOI:** 10.1038/s41431-026-02153-3

**Published:** 2026-07-08

**Authors:** Céline Jost, Alessandro Mussa, Jean-Emmanuel Kurtz, Ashmika Gokhul, Ann Nordgren, Silvia Kalantari, Diana Carli, Andrea Gazzin, Claudio Isella, Enzo Medico, Anna Cassisa, Daniela Cantarella, Thirona Naicker, Antoinette Chateau, Jean-Baptiste Demoulin, Nikolas Herold, Yordi-Michaël Bouhatous, Liubinka Mirakovska, Agnès Maurer, Théo Gaumet, Tara Wenger, Helena Iznardo, Eulalia Baselga, José Manuel Mascaro, Cécilie Bredrup, Olav Aga Kildal, Titas Gladkauskas, Cecilie F. Rustad, Ingrid Voktor Svinvik, Dinel Pond, Elise Schaefer, Maxime Luu, Marc Bardou, Laurence Faivre

**Affiliations:** 1 Université Bourgogne Europe, CHU Dijon Bourgogne, Inserm, CTM UMR1231, équipe GAD, FHU TRANSLAD, Centre de génétique, Centre de référence Anomalies du Développement et Syndromes Malformatifs, Dijon, France; 2 Department of Public Health and Pediatric Sciences, University of Torino, Torino, Italy; 3 Clinical Pediatric Genetics Unit, Regina Margherita Children’s Hospital, Torino, Italy; 4 Département d’hématologie et d’oncologie, Institut de cancérologie Strasbourg Europe (ICANS), Strasbourg, France; 5 Department of Pediatrics and Child Health, Nelson R. Mandela School of Medicine, University of KwaZulu-Natal, Durban, South Africa; 6 Department of Molecular Medicine and Surgery, Center for Molecular Medicine, Karolinska Institutet, Stockholm, Sweden; 7 Department of Clinical Genetics and Genomics, Karolinska University Hospital, Stockholm, Sweden; 8 Department of Clinical Genetics and Genomics, Sahlgrenska University Hospital, Gothenburg, Sweden; 9 Institute of Biomedicine, Department of Laboratory Medicine, University of Gothenburg, Gothenburg, Sweden; 10 Department of Molecular Medicine, University of Pavia, Pavia, Italy; 11 Medical Genetics Unit, Department of Diagnostic Medicine, Fondazione IRCCS Policlinico San Matteo, Pavia, Italy; 12 Department of Oncology, University of Torino, Candiolo, Italy; 13 Candiolo Cancer Institute, FPO-IRCCS, Candiolo, Italy; 14 De Duve Institute, Université Catholique de Louvain, Brussels, Belgium; 15 Division of Pediatric Oncology and Hematology, Department of Women’s and Children’s Health, Karolinska Institutet, Stockholm, Sweden; 16 Pediatric Oncology, Karolinska University Hospital Solna, Stockholm, Sweden; 17 Division of Medical Genetics, University of Washington, Seattle, USA; 18 Department of Dermatology, Hospital de la Santa Creu i Sant Pau, Universitat Autònoma de Barcelona, Barcelona, Spain; 19 Pediatric Dermatology Department, Barcelona Children’s Hospital Sant Joan de Déu, Barcelona, Spain; 20 Department of Ophthalmology, Haukeland University Hospital, Bergen, Norway; 21 Department of Pediatrics, Telemark Hospital, Skien, Norway; 22 Department of Clinical Medicine, University of Bergen, Bergen, Norway; 23 Department of Medical Genetics, Telemark Hospital, Skien, Norway; 24 Department of Pediatrics, Oslo University Hospital, Oslo, Norway; 25 Genetics Clinic, Children’s Hospitals & Clinics of Minnesota, Minneapolis, USA; 26 Department of Medical Genetics, IGMA, Hôpitaux Universitaires de Strasbourg, Strasbourg, France; 27 Université Bourgogne Europe, CHU Dijon Bourgogne, Inserm, CTM UMR1231, équipe GAD, Centre d’Investigations Cliniques Plurithématique 803 (CIC-P 803), Dijon, France

**Keywords:** Molecularly targeted therapy, Genetics, Quality of life

## Abstract

Heterozygous activating variants in platelet-derived growth factor receptor beta (PDGFRβ) are associated with ultra-rare and clinically heterogeneous conditions, including KOGS (Kosaki Overgrowth Syndrome), PS (Penttinen Syndrome) and related conditons. Tyrosine kinase inhibitors (TKIs), which are widely used in hematological and oncological diseases, are now being explored as potential treatments for these conditions. While four published cases have reported encouraging results, there is still insufficient data available to draw definitive conclusions on the benefit–risk ratio. The international consortium Knowing and Treating KOGS/PS was launched to assess the real-life outcomes of such treatments. The consortium presents four new cases and updates four previously published cases of KOGS/PS treated with TKIs from seven countries. A recent publication was also included in the analysis. Individuals received treatment for between three and a half months and eight years (imatinib *N* = 8/8, dasatinib *N* = 3/8, sunitinib *N* = 1/8), with a mean duration of 44.4 months (SD = 29.8). The age at treatment initiation ranged from 6 to 57 years (six patients treated in childhood and two in adulthood). All individuals showed improvement within weeks/months, with minimal side effects. However, efficacy decreased over time in some cases, prompting a switch to a different TKI. These preliminary findings highlight the potential of TKIs for managing KOGS/PS patients. Standardized follow-up protocols and an electronic Case Report Form (eCRF) have been implemented to enhance monitoring and data collection, enabling systematic comparisons between treated and untreated individuals despite the disorders’ rarity.

## Introduction

The syndromes related to a gain-of-function variants in *PDGFRB* include KOGS (#MIM616592), PS (#MIM601812), infantile myofibromatosis (IM) (#MIM228550), and overlapping phenotypes [1]. Although historically described as two distinct entities, the distinction between KOGS and PS is not clinically or biologically relevant, they represent a phenotypic continuum. They will therefore be jointly referred to as KOGS/PS. The molecular basis of KOGS/PS was identified in 2015 through exome sequencing (ES), revealing heterozygous germline activating variants in *PDGFRB*, which encodes the ubiquitously expressed PDGFRβ subunit [2, 3]. Constitutive activation disrupts PDGF-mediated cell proliferation, particularly in mesenchymal tissues. Imatinib, dasatinib, sunitinib, and sorafenib are among the TKIs that inhibit PDGFRβ [4], originally developed for BCR-ABL positive chronic myeloid leukemia (CML) [5, 6] and sunitinib for c-KIT mutation GIST (Gastro Intestinal Stromal Tumor) [1, 7]. In 2016, treatment with a TKI (imatinib) was attempted in a first individual with severe infantile myofibromatosis refractory to conventional chemotherapies [8]. Encouraging results led to TKI use, alone or with vinblastine, in further IM cases, resulting in myofibroma regression with acceptable safety profiles [8–16]. In vitro studies confirmed PDGFRβ inhibition by these TKIs [14–16].

Building on the success of TKIs in IM, their use has been extended to KOGS/PS, with five published cases to date, all showing response to treatment [17–21]. Despite symptom improvement and a favorable safety profile, the available data remain limited due to short follow-up periods (eight weeks to four years), evidence based solely on independent case reports, the use of different TKIs, and the absence of standardized evaluation methods (Table 1). This complicates access to TKIs, as many national health systems require robust evidence before approving off-label treatments. Some clinicians or families also hesitate, given the lack of market authorization and the absence of solid evidence for this indication.

**Table 1:** A: Clinical summary of the four newly treated patients; B: Clinical summary of the five published cases (Follow-up).

A				
	This report Individual 1 France	This report Individual 2 Italy	This report Individual 3 South Africa	This report Individual 4 Sweden
Syndrome	KOGS	KOGS	PS	KOGS
Age at treatment initiation	57 yrs.	6 yrs. 1 m.	6 yrs.	9 yrs. 10 m.
Sex	M	M	F	F
NM_002609.4	c.1751 C > G	c.1696 T > C	c.1997A>G	c.1696 T > C
p.	Pro584Arg	Trp566Arg	Asn666Ser	Trp566Arg
Initial treatment (duration)	Imatinib 22 months	Sunitinib 25 months	Imatinib 6 months Still ongoing	Imatinib 3.5 months, still ongoing
Change of treatment (duration)	Dasatinib 30 months, still ongoing	Imatinib 12 months, still ongoing	Discussion to switch to dasatinib	-
Maximum dose	Imatinib: 400 mg/d Dasatinib: 100 mg/d	Sunitinib: 2.5 mg/d Imatinib: 300 mg/d	Imatinib: 300 mg/d	Imatinib: 170 mg/m^2^/d
Treatment surveillance	Weekly standardized pain scale (VAS); monthly QoL scale SF-36 Weekly, monthly, and then quarterly: Clinical/blood monitoring for safety Quarterly monitoring of the efficacy of treatment (clinical evaluation, GAS scale, six-minute walking test. Annually: data from standard follow-up investigations (when available, bone densitometry, ophthalmology, cerebral and cardiovascular imaging))	Every two months: Clinical/blood monitoring for safety Regular: Evaluating efficacy (clinical evaluation by a clinical geneticist/neuropsychiatrist), six-minute walking test when possible Biannually and then annually due to the stability: head and abdominal MRI for monitoring myofibromas At baseline and at the last follow-up: PedsQL	Two, four, six, eight, and sixteen weeks after the start of treatment, and then monthly: Efficacy monitoring (clinical examination, improvement in activities of daily living, PedsQL), safety monitoring (blood testing, growth monitoring)	Every two weeks: monitor efficacy and adverse effects
In vitro studies	+: switch imatinib > dasatinib	-	+ more sensitive to dasatinib	-
Positive effects	Imatinib: improvement: QoL, spinal pain, walking, no recurrence of pterygium. Plateau effect: change to dasatinib: improvement in pain, walking distance, ability to stand longer, trophic status, QoL. No recurrence of pterygium	Sunitinib: Improved motor performance, OFC/weight ratio, QoL Imatinib: improvement in myofibroma size, QoL, aggressivity, motor functions	Imatinib: More energy, hair regrowth	Imatinib: More energy, improvement in motor performance, peripheral symptoms, appetite, pain, behavior
Side effects	Imatinib: Cutaneous and mucosal (skin dryness) Dasatinib: Transient fatigue and depression, cutaneous and mucosal dryness	Sunitinib: Transient episode of hypoglycemia, increased aggressiveness Imatinib: No side effect reported	Imatinib: Increased CK levels, iron deficiency, anemia, altered taste	Imatinib: Edema, ecchymosis, mild effect on hematologic parameters, decreased growth velocity.

B				
	Pond D et al., 2018 Individual 5 USA	Wenger et al., 2020 LR17-436 Individual 6 USA	Rustad et al., 2021 Individual 7 Norway	Iznardo et al., 2021 Individual 8 Spain
Syndrome	PS	PS	KOGS	PS
Age at treatment initiation	8 yrs. 10 m.	14 yrs.	9 yrs. 6 m.	21 yrs. 3 m
Sex	M	M	M	M
NM_002609.4	c.1996A>C	c.1994T>C	c.1696 T > C	c.1994T>C
p.	Asn666His	Val665Ala	Trp566Arg	Val665Ala
Initial treatment (duration)	Imatinib 8 years Still ongoing	Imatinib 4 years Still ongoing	Imatinib 4 years Terminated after four years but reinstated (see below)	Imatinib Stop: 6 months (Covid-19) Restart: 14 months
Change of treatment (duration)	-	-	Dasatinib was used for less than 8 months, see text for details. Stopped due to adverse effects (restarting imatinib)	Dasatinib 3 years, 8 months, still ongoing
Maximum dose	Imatinib: 400 mg/d	Imatinib: No dose information	Imatinib: 340 mg/m2/d Dasatinib: 100 mg/d	Imatinib: 400 mg/d Dasatinib: 140 mg/d
Treatment surveillance	See Pond et al., 2018	See Wenger et al., 2020	See Rustad et al., 2021	See Iznardo et al., 2021
In vitro studies	+ Use imatinib in first line	NA	+ Variant more sensitive to dasatinib	+: switch imatinib > dasatinib
Positive effects already published	Improvement in contractures, QoL, reduced facial roughness New small stable subcutaneous nodules	Reduction in the visibility of blood vessels, eye irritation, skin sensitivity and turgidity, hand pain, improvement in the appearance of scars, resumption of nail growth	Improved pain, walking distance, sleep, cognition, language skills, social interaction, drooling, fine motor skills, hand grip strength, hair, and scalp trophism	Imatinib: Improvement in hand contractures
New data	Imatinib: At eight years of treatment: clinical stability, plateau effect. Currently administered every 2 days. Change of molecule was discussed (not necessary)	Imatinib: Last news at four years of treatment: no progression of aneurysms and no significant progression of symptoms	Imatinib: At four years of treatment: recurrence of sleep disturbances, joint stiffness and pain. Change to dasatinib: currently four months: adverse effects and no improvement. Discussion to stop dasatinib and restart imatinib	Dasatinib: Improvement in the stiffness and flexibility of the hands, feet, and trunk, mobility
Side effects	Imatinib: Decrease in growth rate	Imatinib: No information on adverse effects	Imatinib: Decreased growth velocity, diffuse oedema, increased CK level, ALAT increased Dasatinib: generalized diffuse erythematous papular rash, hemorrhagic colitis	Imatinib: Transient fatigue Dasatinib: Transient fatigue, increased liver function parameters, mild skin irritation, mild hypertriglyceridemia

*F* Female, *M* Male, *NA* Not Applicable, Goal Attainment Scaling (*GAS*), Quality of Life (*QoL*) scale (Short Form Survey (*SF*-36)) [26, 27], Pediatric Quality of Life Inventory (*PedsQL*) [30], Visual Analogue Scale (*VAS*).

Conventional clinical trials are extremely challenging in ultra-rare diseases like these due to low case numbers, geographic dispersion, clinical heterogeneity, ethical and financial barriers, regulatory hurdles, and limited natural history data. Progress therefore depends on alternative strategies like individual registries, case studies, and real-world evidence. To address this issue, the international consortium “*Knowing and Treating Kosaki/Penttinen Syndromes*” was launched in 2019 and is coordinated by Dijon Bourgogne University Hospital. By 2025, the network includes eighteen teams in thirteen countries and over thirty expert clinicians and researchers (www.kosakipenttinen.org [22]). A real-life observational study on KOGS/PS was initiated, with a particular emphasis on TKI efficacy/safety through standardized assessments. It was approved by the INSERM ethics committee (#23-1042) in France, and registered on ClinicalTrials.gov (NCT05953857) using a dedicated electronic database.

As at mid-2025 [22], nine of 33 patients with KOGS/PS had received TKI treatment (four unpublished cases). Two additional cases were under consideration. Treatment was not started in eight: four due to national regulations and four because individuals or families declined in the absence of formal approval. One mild case with good quality of life did not require therapy. In nine cases, the reasons for no treatment or follow-up were unspecified. Four patients died before treatment could be considered.

These numbers refer only to KOGS/PS individuals with germline *PDGFRB* variants. TKI treatment was also initiated without success in a patient with ocular pterygium–digital keloid dysplasia (OPDKD) [23], with failure attributed to a *PDGFRB* p.(Asn666Tyr) NM_002609.4:c.1996A>T substitution causing markedly increased constitutive autoactivation, particularly at lower temperatures [24]. Among the three known mosaic cases [25, 26], one was treated with sorafenib, though the safety and efficacy data remain unpublished ([26] patient 1).

International efforts are needed for situations where national legislation does not permit compassionate use and for patients/parents awaiting formal approval for treatment. This article presents eight individuals from seven countries (France, Italy, South Africa, USA, Spain, Norway, and Sweden), with four unpublished cases. We explain treatment rationale, procedures, and long-term follow-up before standardized protocols are implemented.

## Patients and methods

Newly included patients were identified through the consortium network, and the authors of previously published cases were contacted to obtain updated long-term follow-up. Individual data were collected via clinician questionnaires covering clinical features, factors influencing treatment initiation, national regulatory frameworks, age at treatment start, dose adjustments, and fibroblast functional testing when needed. Clinicians also reported criteria for treatment efficacy, side effects, TKI switches, overall evolution of benefits and adverse events, and management of plateau effects. Lastly, individual and/or parental satisfaction was reported when assessed. Table 1 detailed the treatment monitoring, clinical descriptions, and outcomes.

### Newly treated individuals

#### Individual 1 (France)

This 61-year-old male, previously reported [27, 28], had normal early development until 15 months when dolichocephaly, tall stature, hyperelasticity, and hyperpigmentation appeared. By age 8, he developed craniofacial features, joint stiffness, scoliosis, atrophic skin, hair fragility, hyperostosis, and pes cavus. Later, recurrent pterygia requiring laser therapy, early osteoporosis, and vascular anomalies occurred, including a basilar artery aneurysm, causing a stroke at age 53. Imaging revealed cerebral artery dolicho-ectasia, arachnoid cysts, Dandy-Walker malformation, and a 16 mm pulmonary nodule consistent with myofibroma. Despite increased functional limitations and fatigue, cognitive function remained normal (IQ 105). Genetic testing identified the pathogenic *PDGFRB* variant p.Pro584Arg NM_002609.4: c.1751 C > G. Due to progressive clinical decline, compassionate use of imatinib was approved following a multidisciplinary review (ICANS, Strasbourg).

#### Individual 2 (Italy)

This nine-year-old male with KOGS had not been previously reported. Prenatal findings showed cerebellar vermis hypoplasia and abnormal tentorium positioning. Born at term via spontaneous vaginal delivery, he had normal birth parameters (weight: 3480 g, length: 52 cm, OFC: 34.5 cm) and adapted well postnatally. Early imaging revealed a posterior fossa arachnoid cyst, enlarged cisterna magna, cerebellar hypoplasia, and progressive macrocephaly, later treated with a ventriculoperitoneal shunt. A ventricular septal defect resolved spontaneously. From four months, facial features appeared (downslanting palpebral fissures, epicanthus, frontal bossing, hypertelorism, broad nasal bridge, thin upper lip) (Suppl. Fig. 1). Multiple histologically confirmed myofibromas developed from six months, including a recurrent orbital lesion. Generalized tonic-clonic seizures began at age two, partially controlled with levetiracetam and clobazam. Neuromotor milestones were delayed (head control at nine months, sitting at twelve months, and assisted walking at three years). Gait was severely limited, requiring continuous support and wheelchair use for longer distances. Ophthalmology noted bilateral corneal leukomas from age three.

At age five, he presented with overgrowth (height 120 cm, + 2.40 SD; weight 21 kg, + 0.83 SD; OFC 53 cm, + 1.40 SD), hyperelastic skin, and joint stiffness. The KOGS diagnosis was confirmed by Sanger sequencing with identification of the heterozygous pathogenic variant *PDGFRB* p.Trp566Arg NM_002609.4: c.1696 T > C. Given the severe phenotype and expected tolerability, compassionate off-label TKI treatment was approved by the ethics committee at age six years.

#### Individual 3 (South Africa)

This previously unreported six-year-old female was abandoned at birth and experienced early malnutrition. Initial evaluation at age five years ten months revealed global developmental delay with normal growth parameters (height 110 cm (-0,72 SD), weight 22 kg ( + 0.75 SD), OFC unavailable). Clinical features included facial features (long face, large forehead, hypertelorism, downslanting palpebral fissures, midface hypoplasia, prognathism), skeletal anomalies (pectus excavatum, large hands, square fingers, fixed flexion deformities of her fingers, elbows, legs), and significant skin fragility with dystrophic scars and nodules consistent with juvenile hyaline fibromatosis, severely impacting daily activities. Imaging identified a large posterior fossa arachnoid cyst, craniosynostosis, and lung fibrosis. A p.Asn666Ser NM_002609.4: c.1997A>G variant in the *PDGFRB* gene was found by ES, confirming the diagnosis of PS. Given the severity of the phenotype, compassionate use of a TKI was approved, pending the approval of medical aid. Imatinib was the only drug available in South Africa, and the cheaper generic was used. In vitro cellular studies on the individual’s fibroblasts to evaluate the best TKI were also conducted.

#### Individual 4 (Sweden)

This previously unreported female aged nine years, ten months, was born to Swedish-Norwegian parents. The father had epilepsy diagnosed at age twenty. The pregnancy was uneventful until 42 weeks, when a prenatal ultrasound identified a retrocerebellar cyst, prompting delivery by C-section at 42 + 1 weeks of gestation. Birth parameters were normal (weight: 3330 g (37th centile), length: 51 cm (59th centile), OFC: 35 cm (58th centile)). Postnatally, a retrocerebellar cyst and arthrogryposis (elbow and knee contractures) were noted, cyst fenestration at seven weeks was followed by hydrocephalus treated with a ventriculoperitoneal shunt at nine weeks. Early development was normal. Seizures began at fifteen months with abnormal sleep EEG (spike/spike-wave complexes). At two years, absence, febrile (twenty minutes), and focal seizures occurred. Levetiracetam at four years achieved control. Motor difficulties (weakness, stiffness, fatigue) and hip, back, and legs pain developed later, limiting mobility. She presented with tall stature, joint stiffness, distinct facial features (brachycephaly, broad nasal root, prominent supraorbital ridges, thin upper lip, low-set right ear), bilateral optic atrophy, visual impairment, hyperelastic pale skin, lipoma, hemangiomas, and cold-induced vasospasms. At age three, trio genome sequencing (GS) identified a heterozygous de novo *PDGFRB* variant: p.(Trp566Arg) NM_002609.4: c.1696 T > C, confirming KOGS. Between ages eight and nine, rapidly progressive scoliosis required spinal surgery. Cardiovascular evaluation revealed mild coronary artery dilation managed with prophylactic acetylsalicyl acid. Neurovascular imaging showed mildly increased perivascular spaces but no aneurysms. Constipation and recurrent urinary infections occurred without renal abnormalities. Autism spectrum disorder was diagnosed with sensory hypersensitivity. Growth has been consistent (height +2.5 SD, weight +1 SD, OFC + 1 SD). Compassionate imatinib treatment was approved, given the phenotype’s severity (coronary dilation, scoliosis, chronic pain).

### Updating the follow-up of published cases of individuals treated with TKIs

The procedures for initiating and monitoring treatment are detailed in their respective articles [18–20, 24] (summary in Table 1). No follow-up data were available for the patient reported in the 2025 publication [21].

### In vitro TKIs testing

Supplemental Materials.

**Fig. 1:**
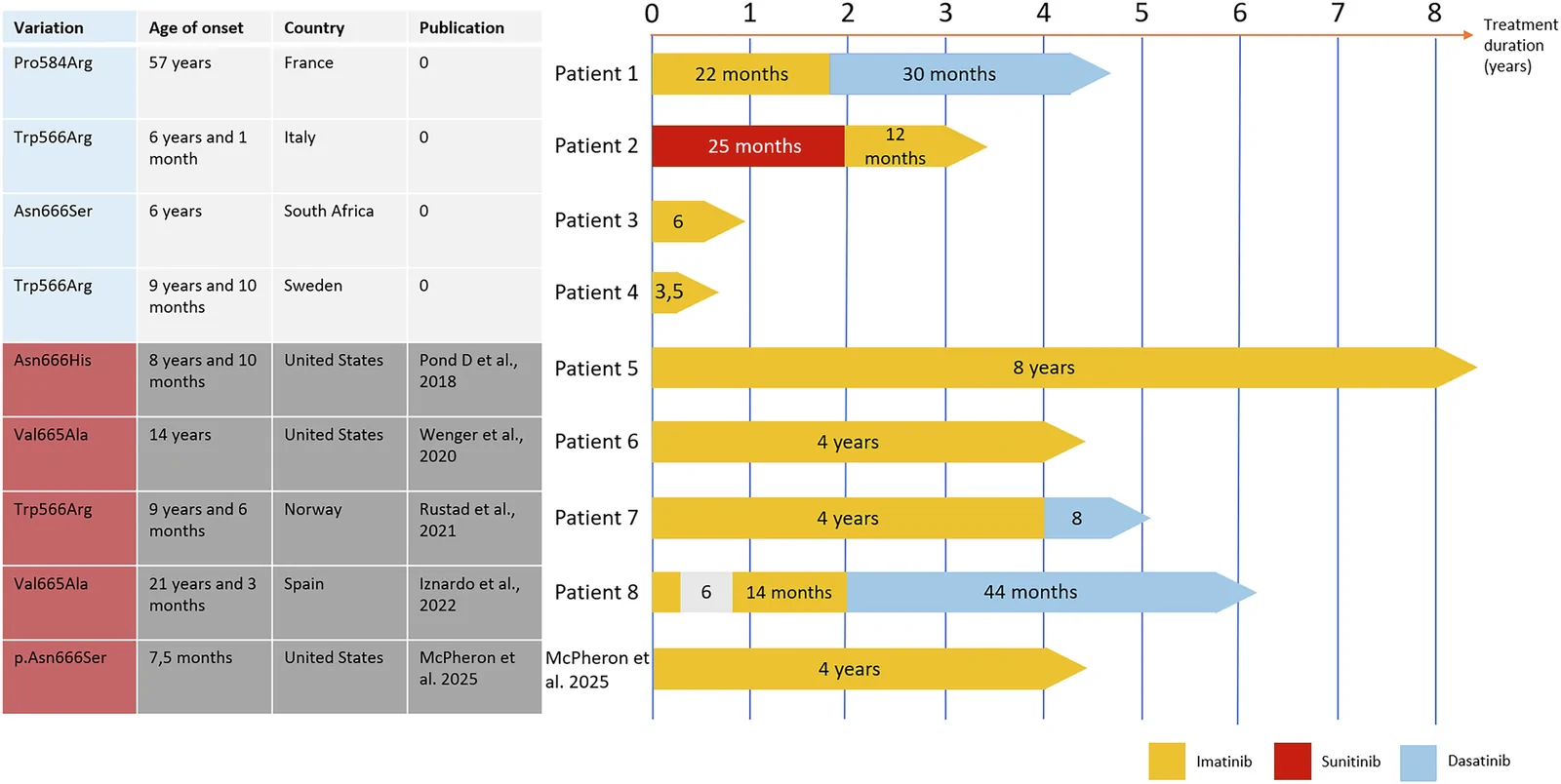
Overview of TKI treatment strategies and duration in eight individuals with KOGS/PS. **Left panel:** Genetic variant, age at treatment initiation, country, and reference of publication for each individual. Newly reported cases are indicated without prior publication, whereas previously published cases are referenced accordingly. **Right panel:** Graphical representation of treatment duration (in years) and sequencing of TKIs for each patient. Bars illustrate cumulative exposure and treatment switches over time. Yellow indicates imatinib, red indicates sunitinib, and blue indicates dasatinib, grey indicates treatment interruptions or drug holidays. Arrowheads denote ongoing treatment at last follow-up. Durations are shown in months or years as specified within each bar.

## Results

### Newly treated individuals (Fig. 1)

#### Individual 1 (p.Pro584Arg NM_002609.4: c.1751 C > G): France

**Fig. 2:**
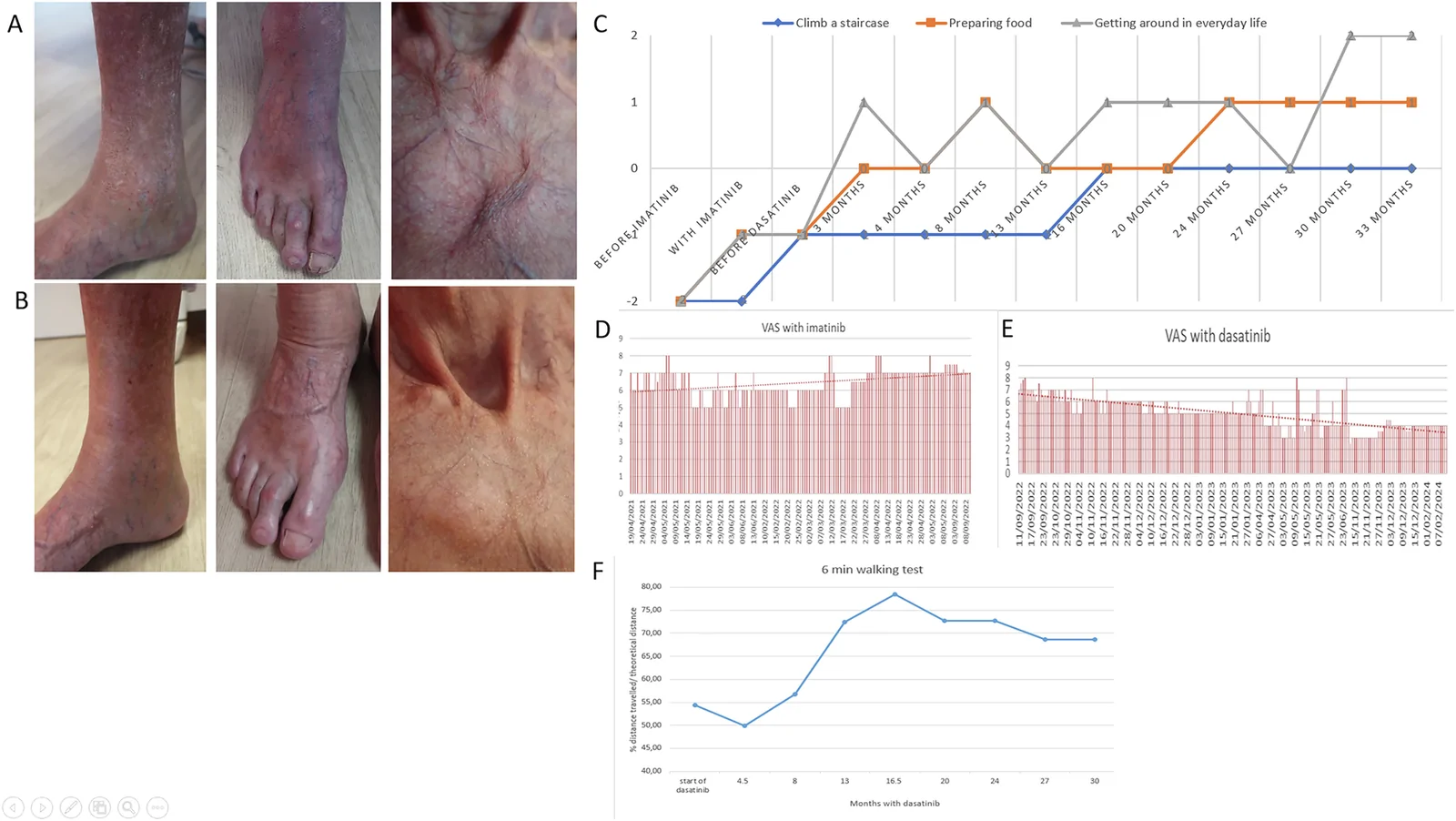
Clinical follow-up of Individua1. **A** Skin appearance before treatment with dasatinib: note hyperpigmentation, atrophic and scaly skin, high visibility of the venous network, adhesion of the skin, hypertrophic scars, and brittleness of the nails. **B** Skin appearance after treatment: note the improvement in skin trophicity, as well as in scars and nails, was observed, with resolution of scaling and reduced visibility of superficial veins., **C** GAS evaluations under imatinib and dasatinib. **D** VAS under imatinib. **E** VAS under dasatinib. **F** Distance covered during the six-minute walk test under dasatinib.

Imatinib was initiated at age 57 (100 mg/day) and gradually increased to 400 mg/day. Within two months, the individual reported 30% less spinal pain, improved walking/mobility, and no recurrence of corneal abnormalities previously needing regular laser treatments. Pain scores (VAS) (Fig. 2D) and quality-of-life measurements (SF-36) showed minimal changes, and skin and orthopedic issues remained stable. Clinical tolerance was good except for minor skin dryness. After one year, efficacy declined significantly, and the individual reported losing around 35% of the progress he had made. In vitro studies of patient fibroblasts showed that dasatinib had the strongest inhibitory effect on PDGFRβ (Suppl. Fig. 2) After 22 months, treatment was switched to dasatinib (50 mg/day, increased to 100 mg/day). Dasatinib significantly improved his standing duration and skin condition (Fig. 2A, B), reduced bruising and bleeding, enhanced sexual function, and prevented the recurrence of pterygium. Pain decreased notably (VAS reduced from 7 to 3–4 overall, and from 7–8 to 1–2 in the mornings) (Fig. 2E). He achieved and surpassed his goals on the GAS scale (Fig. 2C), though SF-36 remained unchanged. Walking improved from 54% to 78% of the theoretical distance in the first sixteen months, then stabilized (Fig. 2F). Gait, flexibility, scoliosis, vascular anomalies, and pulmonary nodules remained stable. Since initiation of TKI therapy, his dentist has also reported markedly reduced gingival bleeding and a substantial decrease in dental caries, allowing longer intervals between dental follow-up visits.

**Table:** 

Climbing a staircase	
−2 (Much worse than target)	I can only climb 1 floor.
−1 (Initial state)	I can climb a maximum of 2 floors without difficulty.
0 (The target)	I can climb 3 to 5 flights of stairs despite my shortness of breath and moderate pain.
+1 (Slightly better than target)	I can climb 6 to 7 flights of stairs with a feeling of breathlessness and slight pain.
+2 (Much better than target)	I can climb stairs without any limitations or difficulties.
Preparing food	
−2 (Much worse than target)	I spend less than 15 minutes in the kitchen preparing my meals.
−1 (Initial state)	I can spend 15 minutes in the kitchen preparing my meals.
0 (The target)	I can spend 20 to 30 minutes in the kitchen preparing my meals.
+1 (Slightly better than target)	I can spend 30 to 45 minutes in the kitchen preparing my meals.
+2 (Much better than target)	I don’t feel I have a time limit for preparing my meals.
Getting around in everyday life	
−2 (Much worse than target)	I leave my home less than 2 times a week.
-1 (Initial state)	I go out of the house 3 (or even 4) times a week.
0 (The target)	I leave my home 5 to 6 times a week.
+1 (Slightly better than target)	I go out of the house 1 time a day, i.e., 7 times a week.
+2 (Much better than target)	I can leave my home several times a day, i.e., more than 7 times a week.

Early mild skin and mucosal side effects resolved spontaneously within six months. Fatigue, insomnia, and mood fluctuations persisted.

The patient considered dasatinib more effective and sustained and stated: “I would have preferred to receive TKI treatment earlier, before many complications had become established.”

#### Individual 2 (p.Trp566Arg NM_002609.4: c.1696 T > C) : Italy

**Fig. 3:**
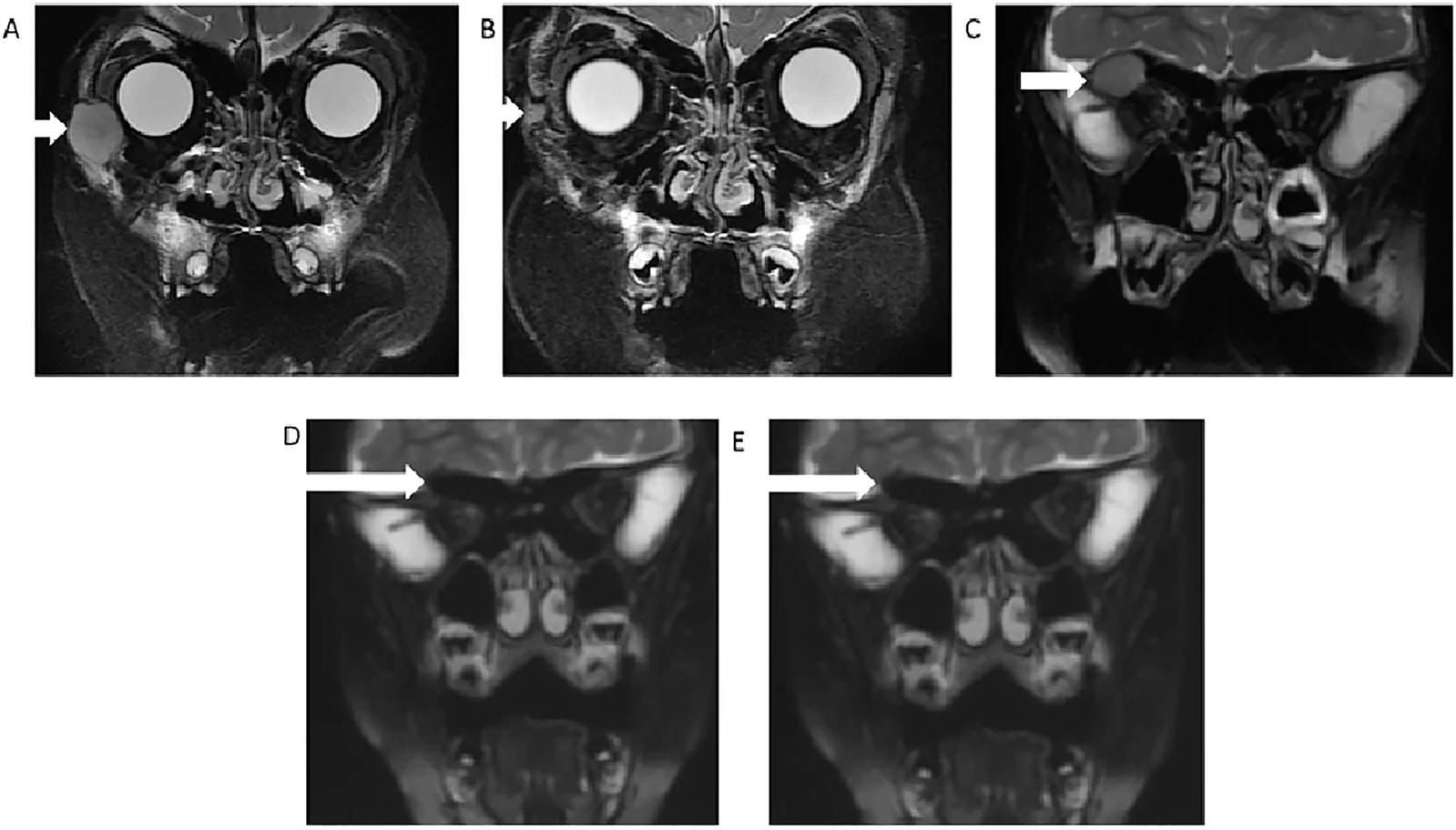
MRI of Individual 2: evolution of right orbital myofibroma before and under treatment. **A** Arrow : Right orbital myofibroma before treatment, dimensions approximately 20 × 13 mm 33 months before the therapy started. **B** Arrow : Myofibroma after surgery alone 26 months before the therapy started. **C** Arrow: Myofibroma relapse 18 months post-surgery, 11 months before the therapy started. **D**. Arrow : Myofibroma after 7 months of sunitinib. **E** Arrow: Myofibroma after 35 months of TKIs.

Sunitinib was initiated at 12.5 mg/day at age six years. Within six months, previously uncontrolled tonic-clonic seizures ceased completely. Motor skills improved significantly, enabling walking over 100 meters with minimal support. Most myofibromas remained stable, but the recurrent right orbital myofibroma resolved on MRI after seven months of starting therapy (Fig. 3D). Body proportions changed slightly during treatment: OFC/height ratio decreased from 0.42 at baseline to 0.39 after two years, and OFC/weight ratio from 2.16 to 1.92. Mild transient asymptomatic hypoglycemia (47 mg/dL) occurred. During TKI therapy, no other alteration was found in the monitoring blood analysis. After 25 months, owing to a partial myofibroma response, apart from the orbital lesion which showed regression, and following collegial discussion within the consortium, treatment was switched to imatinib (300 mg/day). Six months later, modest regression in the remaining myofibromas was observed at evaluation visits. The parents noted behavioral improvements, particularly reduced aggression episodes. Motor function further improved, enabling short-distance walking independently. Quality-of-life scores (PedsQL) significantly improved from baseline (age 6 years) to the last follow-up (age 9 years): physical functioning (31.5–46.9), social functioning (20–35), school functioning (30–40), and total score (33.7 –43.5). Only emotional functioning showed a slight decrease (55–50), Social difficulties complicated assessments.

After approximately three years of TKI treatment, no relapse of the right orbital myofibroma was evident on brain MRI (Fig. 2E). No notable changes in skin or joint flexibility were observed.

#### Individual 3 (p.Asn666Ser NM_002609.4: c.1997A>G): South Africa

**Fig. 4:**
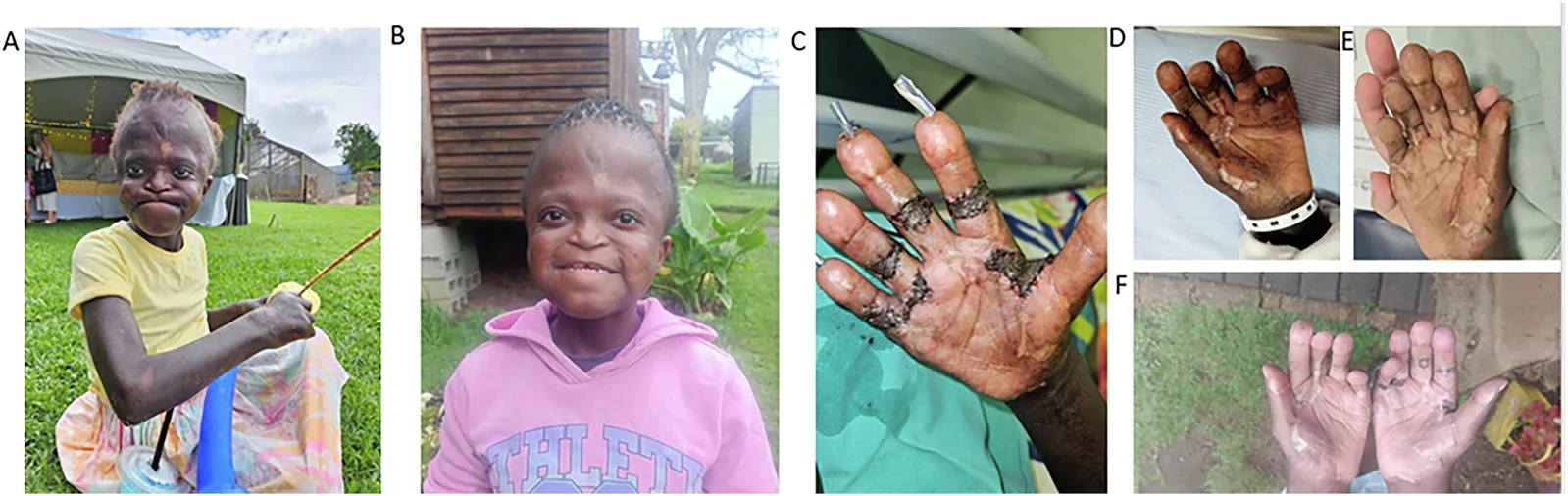
Follow-up photographs for Individual 3. **A** Face photographs before treatment: Note the long face, large forehead, hypertelorism, down-slanting palpebral fissures, midface hypoplasia, large tongue, prognathism), skin lesions (multiple dystrophic scaring, areas of atrophy) and sparse hair. **B** Face photographs after six months of treatment: Note the improvement in overall skin appearance, with scars and areas of atrophy less visible, overall lighter skin and less sparse hair. **C** Hands photographs: appearance of hands immediately after surgery (July 2024). **D** Hands photographs: appearance of hands after approximately 1 months post-surgery, with treatment. **E** Hands photographs : appearance of hands after approximately 2 months post-surgery, with treatment. **F** Hands photographs: appearance of hands after approximately 4 months post-surgery, with treatment.

Imatinib was started at age 6 (150 mg/day), increased to 300 mg/day after one month. The patient and parents reported increased energy and improved overall well-being. Hair regrowth occurred, but keloid lesions remained unchanged (Fig. 4A, B). Surgery was performed to release hand contractures, and postoperative new flexion deformities gradually improved with continued therapy (Fig. 4C–F). Mild adverse effects included elevated CK (319 IU/L), iron deficiency (6.6 µmol/L), anemia, and transient altered taste, which quickly resolved. At the last follow-up, six months after treatment initiation, clinical improvement was ongoing. In vitro fibroblast studies to evaluate the best TKI evidenced that imatinib, axitinib, and dasatinib effectively inhibited the *PDGFRB* p.Asn666Ser NM_002609.4:c.1997A>G variant, with dasatinib demonstrating the most potent inhibitory effect (Supplemental Fig. 3).

#### Individual 4 (p.Trp566Arg NM_002609.4:c.1696 T > C.): Sweden

Imatinib was started at age nine years ten months (170 mg/m²). After one month, her energy and motivation increased markedly, and she could walk five km with enthusiasm. Raynaud-like symptoms in the toes improved. Two weeks after initiation, mild superficial ecchymoses ( ≤ 2 cm) on her extremities and limb edema developed. Due to severe autism spectrum disorder, blood monitoring was not feasible. Given the sustained clinical benefit and mild adverse effects, treatment was continued at half dose.

At the most recent follow-up, conducted 3.5 months after initiation of imatinib, the patient demonstrated marked clinical improvement. Peripheral symptoms had improved, with toes no longer as cold or cyanotic. Appetite was improved, and pain levels were reduced. Behavioral status had improved; whereas she had previously not tolerated physical contact, including hugs or clinical examination, she was now able to accept them. Her growth velocity decreased. The treatment was well tolerated, with only mild effects on hematologic parameters. She experienced a single self-limiting episode of epistaxis and continues to display an increased tendency toward hematoma formation.

### Updating previously published data (Table 1)

#### Individual 5: Pond *et al*., 2018 [20] (p.Asn666His NM_002609.4: c.1996A>C): USA

This first KOGS/PS patient began imatinib at age eight years, ten months (400 mg/day) after in vitro testing confirmed its efficacy. After one year, he showed significant improvement in contractures, quality of life, and facial roughness. Minor side effects resolved spontaneously. A slight decrease in his growth rate led to a dose reduction to 400 mg every two days.

Over nine years of treatment, the clinical benefits persisted. Due to concerns about growth restriction and long-term toxicity, the family progressively reduced the dose (200 mg/day, then 150 mg/day, and later 100 mg/day). A drug holiday at age twelve caused rapid swelling recurrence in his hands and feet within three days. Switching to generic imatinib repeatedly triggered swelling or discomfort, especially in his hands.

Multiple subcutaneous nodules, present from the treatment start, increased in number but remain stable in size and asymptomatic, they have not been biopsied. No TKI switch has been attempted. At age seventeen, his height was 163 cm; a conjunctival cyst appeared at sixteen years with possible surgery planned. At the last follow-up, he had graduated high school and planned to attend college. Growth parameters: height 190.5 cm (above the mid-parental estimate of 182 cm) and weight 81.6 kg. Currently, he takes 100 mg every 2–3 days or when swelling appears. Clinical follow-up did not report any evidence of pubertal delay during treatment.

#### Individual 6: Wenger *et al*. 2020 [17] (p.Val665Ala NM_002609.4:c.1994T>C): USA

This adolescent with PS, previously reported by Johnston et al. [3] as Individual 3, began imatinib at age fourteen. After one month, he showed improved skin turgor, reduced vessel visibility, decreased skin sensitivity, reduced hand pain, and improvement in ocular discomfort and conjunctival injection. By two months, his scars were thinner and less prominent, and nail growth had resumed [17]. At age eighteen, after four years of imatinib treatment, he remained clinically stable with no significant progression of symptoms and no progression of aneurysms on imaging, an unexpected finding for PS.

#### Individual 7: Rustad *et al*. 2021 (p.Trp566Arg NM_002609.4: c.1696 T > C) [19]: Norway

This patient [19] began imatinib at age nine years six months after in vitro testing confirmed sensitivity to imatinib. Initial benefits included reduced pain and cramps, improved sleep, mobility, cognition, speech, social interaction, decreased drooling, and better scalp/hair health. Grip strength increased. No significant side effects occurred in the first eighteen months.

After four years of imatinib (340 mg/m^2^), some symptoms recurred (joint pain, stiffness, disturbed sleep), improved by short-term analgesics. Neurological function remained stable. Hair, initially thicker, thinned in the frontal region. Growth rate decreased from the 99th percentile (151 cm at 9.5 years) to the 38th percentile (170 cm at 15 years). Head circumference remained at the 100th percentile, and brain MRI after 3.5 years showed no change. In 2022, pleural effusions due to bacterial pneumonia required drainage. Persistent pitting edema limited dose increases.

Due to declining efficacy, imatinib was discontinued after four years and replaced with dasatinib (up to 100 mg/day). The patient intermittently required analgesics and appeared somewhat stiffer, but experienced improved growth, that could also be due to the pubertal growth spurt. According to the available clinical records, puberty reportedly began at approximately 9.5 years of age, and no pubertal delay was noted. The patient is currently growing along the 38th centile, although precise length measurements are challenging due to significant contractures. Neurological status and edema remained unchanged. After seven weeks, a generalized erythematous papular rash appeared. At ten weeks, bloody diarrhea, hypoalbuminemia, and elevated calprotectin developed with negative infectious workup. Symptoms resolved after reducing dasatinib (50 mg/day) and iron supplementation. Gradual re-escalation to 100 mg/day caused the recurrence of bloody stools, prompting discontinuation after three months. Imatinib is now being restarted.

#### Individual 8: Iznardo *et al*. 2021 [18] (p.Val665Ala NM_002609.4: c.1994T>C): Spain

This patient began imatinib at 21 years 3 months (200 mg/day, increased to 400 mg/day after 3 weeks due to the absence of adverse effects). After sixteen months, improvement was limited to reduced hand contractures with no major side effects. In vitro studies indicated greater sensitivity to dasatinib, prompting a switch (70 mg/day, escalated to 140 mg/day) [18].

After one month on dasatinib, there was further reduction in flexion contractures and skin induration, with improved range of motion in the hands and feet. The patient gained notable flexibility in the hands, feet, and trunk, performing movements that were previously impossible (push-ups, sit-ups). Over three years, eight months of follow-up at 140 mg/day, these benefits were sustained. P-ROM scores improved from Shoulder 5/7, Elbow 6/7, Wrist-hands 4/7 (baseline post-imatinib) to Shoulder 6/7, Elbow 6/7, Wrist-hands 5/7 after three years two months. Side effects have been minimal: mild skin irritation, slight increase in liver function tests, and mild hypertriglyceridemia.

## Discussion

This study is the first to report long-term follow-up data, up to nine years, on eight individuals with KOGS/PS treated with TKIs, including four new cases and updates on four previously published ones. A ninth patient reported in a recent publication was also considered [21]. Most showed improvement or disease with treatment, with no new safety concerns [17, 18, 20, 21, 24]. The eight individuals began treatment at a median age of 9.7 years (range 6 –57 years). Imatinib was the first-line treatment for all except one who received sunitinib. Four individuals switched treatment, three from imatinib to dasatinib and one from sunitinib to imatinib, while two are in the process of switching from imatinib to dasatinib, and one will return to imatinib due to adverse effects caused by dasatinib. The median treatment duration with TKIs was 50 months (range 3.5 month–8 years), with imatinib at 14 months (3.5 month–8 years), dasatinib 30 months (4 months- 3 years), and one individual receiving sunitinib for 25 months. Dosing was managed by experienced oncologists/hematologists with gradual escalation to standard CML targets according to tolerance. Defining a single endpoint for treatment efficacy is challenging due to phenotypic heterogeneity, age, disease progression, and personal expectations. Individual 1 hoped for functional gains (walking/mobility, endurance, fatigue, pain), which were achieved. He was surprised by improved skin fragility and no pterygium recurrence. For his physicians, a key aim was preventing vascular complications, a major cause of morbidity/mortality in KOGS/PS. Individual 2, with syndromic myofibromas, mainly sought their reduction. Individual 3 aimed to improve joint limits and keloid scarring; additional benefits included increased muscle tone, higher energy, and hair regrowth. Individual 4 targeted scoliosis, leg pain, and coronary dilation; energy improved markedly after one month. Overall, reported benefits were heterogeneous: six showed better motor performance, six noted improvements in their skin, hair, or nails, and some described an improvement in pain, flexibility/contracture, and ocular anomalies. Yet the common theme was enhanced general well-being in all individuals. Given the clinical heterogeneity and differing expectations, patient-reported outcome measures (PROMs), such as the Goal Attainment Scale (GAS) [29, 30], appear particularly appropriate for assessing treatment response, according to the individual’s baseline expectations. Used for Individual 1, the GAS seemed more suitable than QoL scales, which proved challenging to apply to a condition with fluctuating symptoms. Faced with this naturally progressive disease, we also need to ask what can be considered a positive effect of treatment. In such cases, clinical stabilization may itself represent a significant outcome. Indeed, the expected natural history of KOGS/PS is characterized by gradual progression of connective tissue abnormalities, increasing joint contractures, progressive vascular involvement including aneurysmal changes, and cumulative functional decline over time. In untreated individuals reported in the literature, musculoskeletal stiffness, skin induration, and vascular complications typically worsen with age. Therefore, stabilization or even modest improvement may represent a meaningful deviation from the anticipated disease trajectory rather than mere fluctuation. Explicit consideration of the natural course is therefore essential when interpreting therapeutic benefit.

Efficacy sometimes declined but not systematically, depending on the TKI. Newly reported cases were switched or planned to switch to second-line TKIs. For individuals 1 and 3, dasatinib replaced imatinib due to suboptimal responses. Long-term follow-up (up to nine years [20]) also showed reduced efficacy in most, with two switching TKIs (one guided by fibroblast testing [18, 19]). Dasatinib, rarely first-line, is often used second-line. To date, although this should be interpreted with caution, no decline in dasatinib’s effectiveness has been observed. This mirrors findings in CML, where around 20% of imatinib responders eventually lose response. Switching to a second-generation TKI like dasatinib, or increasing imatinib dosage, is a common strategy [31]. Differences in efficacy may relate to target profiles: imatinib (BCR-ABL, c-KIT, CSF-1R, PDGFRα/β), dasatinib (BCR-ABL, SRC family kinases, c-KIT, EPH, PDGFRβ), and sunitinib (c-KIT, PDGFRα/β, VEGFR, FLT3, RET, CSF-1R) [32]. These differences are relevant for drug selection and supported by known PDGFRβ activation and kinase inhibition spectra [2, 3, 33–35]. Notably, loss of efficacy is unreported in IM. Disease progression during TKI therapy may not necessarily reflect treatment failure or reduced drug sensitivity, but rather the natural course of a chronic, progressive condition.[8–16]. Fibroblast testing was used in selected cases to compare ex vivo sensitivity to different TKIs and helped inform therapeutic switching decisions in situations of suboptimal response.

In this context, a recent in vitro study systematically demonstrated variant-dependent differences in TKI sensitivity across recurrent activating *PDGFRB* substitutions [36]. In our cohort, treatment initiation or switching was, in five of eight cases, informed by functional studies performed on patient-derived fibroblasts, allowing in vitro comparison of inhibitor efficacy for the specific variant involved. Our long-term clinical follow-up provides a complementary dimension to these in vitro findings, as only longitudinal observation can assess durability of response, the impact on progressive manifestations, and real-life tolerability. The concordance observed in several individuals between fibroblast sensitivity testing and clinical evolution supports a translational, genotype-informed approach to therapeutic decision-making in *PDGFRB*-related disorders.

These observations also suggest that functional testing on patient-derived fibroblasts could potentially be performed before treatment initiation in order to help guide individualized TKI selection and optimize therapeutic management from the start. Nevertheless, such assays remain exploratory and are not standardized for routine clinical decision-making. Variability in culture conditions, readouts of downstream signaling, and the still-limited correlation between ex vivo inhibition and in vivo clinical response could represent important limitations. In addition, for several recurrent variants, sensitivity data may already be available from previously published studies, potentially limiting the need for systematic patient-specific testing. Further harmonization of functional assays may strengthen their translational applicability and future integration into clinical practice.

Despite promising results, benefit–risk assessment remains difficult due to the ultra-rare nature of KOGS/PS, limited natural history data, and trial challenges. To address this, the consortium created an eCRF for standardized monitoring of treated/untreated patients to enable real-life comparisons. Earlier treatment was hypothesized to improve outcomes, and current data (six started before adulthood, two after) showed that all responders, regardless of age, display an initial strong response followed by more modest effects. Future eCRF data may clarify the role of early intervention.

Side effects varied between individuals, but most tolerated treatment well, similar to CML and GIST. Serious imatinib-related events in CML are rare and occur mainly in the first year. After five years, no late toxic effects were seen, supporting long-term safety [7]. In this study, most adverse events (skin symptoms, mild laboratory changes) resolved spontaneously. Only two led to dose change/cessation: growth rate deceleration (Individual 4 [20]) and bloody diarrhea, a known dasatinib side effect (Individual 7, dasatinib [19]), prompting a switch back to imatinib. TKI are associated with a spectrum of class-related adverse effects that require careful clinical monitoring during treatment. The most frequently reported toxicities include hematologic abnormalities (such as anemia, neutropenia, or thrombocytopenia), fluid retention and edema, gastrointestinal symptoms, dermatologic reactions, fatigue, and biochemical abnormalities, including elevations in liver enzymes. Awareness of these commonly reported categories of adverse events is important for appropriate clinical follow-up during long-term therapy [37].

In conclusion, these new data contribute to the growing body of literature supporting, when possible, the value of the compassionate use of TKIs for KOGS/PS individuals, with to date, a satisfying long-term safety profile. Healthcare professionals must remain aware that, in this naturally progressive and severe disease, stabilizing the condition could also be considered as success. The consortium’s initiative to standardize the follow-up of treated and untreated patients will make it possible to conduct the most robust evaluation possible in a real-life setting and overcome the challenge of formal clinical trials in ultra-rare diseases.

## Supplementary information

Supplemental data

supplemental legendes

Supplemental figure 1

Supplemental figure 2

Supplemental figure 3

## Data Availability

The datasets generated and/or analysed during the current study are available from the corresponding author on reasonable request.
