# Supplementary material for: Tyrosine kinase inhibitors in Kosaki/Penttinen syndromes: new reports, follow-up of treated individuals and literature review
Source: Eur J Hum Genet. 2026 Jul 8;34(10):1454–64. doi: 10.1038/s41431-026-02153-3 (PMC13634052; doi:10.1038/s41431-026-02153-3)
Supplement: Supplementary file 1 [file 41431_2026_2153_MOESM1_ESM.docx]

***Supplemental Materials and methods (functional testing individual 1 and 3)***
**Cell culture (Norwegian Team)** ^1,2^

Patient fibroblasts were seeded in 10cm petri dishes. When the cells were 80% confluent, they were serum-starved for six hours. Cells were harvested in 1% NP-40, 20 mM Tris (pH 8.0), 137 mM NaCl, 10% glycerol, 2 mM EDTA, 1 mM activated sodium orthovanadate, 10 μg/ml aprotinin (#4139, Tocris, Abington, United Kingdom), and 10 μg/ml leupeptin (leupeptin hemisulfate, #1167, Tocris, Abington, United Kingdom), and stored at -80^o^C until immunoblot or ELISA analysis was performed.

**Treatment with Tyrosine Kinase Inhibitors *in vitro***

Six different TKIs were used for treatment: with axitinib (AG 013746), dasatinib (BMS-354825), imatinib (STI571), sunitinib (SU11248), nintedanib (S1010), and sorafenib (S7397) (all Selleckchem, Munich, Germany). Three concentrations (0.1EC, 1EC, and 10EC) were used as provided by the manufacturer. Patient fibroblasts harboring the p.Pro584Arg *PDGFRB* variant for individual 1 and the p.Asn666Ser *PDGFRB* variant for individual 3 were serum-starved for six hours and treated with TKIs for four hours prior to harvesting. Controls were kept untreated with TKIs.

**ELISA and immunoblot analysis**

ELISA analysis was conducted using a DuoSet® IC PDGFRβ kit (# DYC1767-2, R&D Systems, Minneapolis, MN) following the guidelines provided by the manufacturer. For immunoblot analysis we used primary antibodies from Cell Signaling Technology (Danvers, MA) at recommended dilutions (catalog numbers in brackets): phospho-Ser473-AKT (#4060), AKT (#4691), phospho-Tyr783-PLCγ1 (#2821), PLCγ1 (#5690), anti-rabbit IgG, and HRP-linked antibody (#7074). To control for equal loading, an anti-GAPDH antibody (#G99545, Sigma-Aldrich) was used.

1.	Bredrup C, Stokowy T, McGaughran J, et al. A tyrosine kinase-activating variant Asn666Ser in PDGFRB causes a progeria-like condition in the severe end of Penttinen syndrome. *Eur J Hum Genet EJHG*. 2019;27(4):574-581. doi:10.1038/s41431-018-0323-z

2.	Bredrup C, Cristea I, Safieh LA, et al. Temperature-dependent autoactivation associated with clinical variability of PDGFRB Asn666 substitutions. *Hum Mol Genet*. 2021;30(1):72-77. doi:10.1093/hmg/ddab014

3.	Iznardo H, Bredrup C, Bernal S, et al. Clinical and molecular response to dasatinib in an adult patient with Penttinen syndrome. *Am J Med Genet A*. 2022;188(4):1233-1238. doi:10.1002/ajmg.a.62603
