# Supplementary material for: Tyrosine kinase inhibitors in Kosaki/Penttinen syndromes: new reports, follow-up of treated individuals and literature review
Source: Eur J Hum Genet. 2026 Jul 8;34(10):1454–64. doi: 10.1038/s41431-026-02153-3 (PMC13634052; doi:10.1038/s41431-026-02153-3)
Supplement: Supplementary file 2 [file 41431_2026_2153_MOESM2_ESM.docx]

**Supplemental Figure 1 :** Clinical photographs of Individual 2, highlighting the evolution of facial features and hand/foot anomalies over time

A. A-D: 4 months;
B. E: 14 months;
C. F,I: 5 years 2 months;
D. G,H,J: 7 years 3 months of age. Clinical features became more pronounced over time, including a prominent forehead, downslanting palpebral fissures, broad and high nasal bridge, thin upper lip, and pointed chin.
E. K: Hyperelastic and thin skin in the thoracic region, with myofibroma (arrow).

**Supplemental Figure 2 :**

Supplemental Figure 2. Individual 1 : **In vitro tyrosine kinase inhibitor sensitivity testing in patient-derived skin fibroblasts carrying PDGFRB p.Pro584Arg**

A: Effects of tyrosine kinase inhibitors (TKIs: imatinib, dasatinib, axitinib, sunitinib, nintedanib, sorafenib) on PDGFRβ phosphorylation in patient fibroblasts carrying the p.Pro584Arg PDGFRB variant. TKIs were tested at 0.1EC, 1EC, and 10EC concentrations (as detailed in Supplemental Materials). Statistical significance was evaluated using one-way ANOVA followed by Dunnett's multiple comparisons test. ****P < 0.0001, ***P < 0.001, **P < 0.01.

B: Total phosphorylated PDGFRβ levels in serum-starved fibroblasts from two healthy controls (CTRL1, CTRL2) and a patient harboring the p.Pro584Arg PDGFRB variant (KOGS). Data are presented as mean ± SEM from three independent experiments. Statistical analysis was performed using one-way ANOVA followed by Dunnett's multiple comparisons test. ****P < 0.0001.

**C.** Immunoblotting corresponding to Figure 1 Panel A.

**D.** Quantification of blots corresponding to Figure 1 Panel A.

Table summarizing the EC50 concentrations of tyrosine kinase inhibitors (TKIs) for PDGFRβ as provided by the manufacturer (Selleckchem). The values represent the effective concentration (nM) required to inhibit 50% of PDGFRβ activity. * For dasatinib, the manufacturer does not provide an EC50 for PDGFRβ but indicates it targets Abl, Src, and c-Kit with EC50 values of <1 nM, 0.8 nM, and 79 nM, respectively. This study utilized a concentration of 100 nM dasatinib as previously described (18).

| Tyrosine Kinase Inhibitor (TKI) | EC50 (Manufacturer-Provided, nM) |
| --- | --- |
| Imatinib | 100 |
| Dasatinib | 100***** |
| Axitinib | 1.6 |
| Sunitinib | 2 |
| Nintedanib | 65 |
| Sorafenib | 57 |

Although PDGFRβ signaling also activates downstream MAPK pathways, ERK and phosphorylated ERK (pERK) levels were not systematically assessed in this study. Therefore, potential differential regulation or compensatory activation of the MAPK pathway in response to TKI-mediated PI3K–AKT inhibition cannot be excluded and warrants further investigation.

**Supplemental Figure 3. Individual 3 : In vitro tyrosine kinase inhibitor sensitivity testing in patient-derived skin fibroblasts carrying PDGFRB p.(Asn666Ser) variants.**

Effects of tyrosine kinase inhibitors (TKIs: imatinib, dasatinib, axitinib, sunitinib) on PDGFRβ phosphorylation in patient fibroblasts carrying the p.Asn666Ser PDGFRB variant. Statistical significance was evaluated using one-way ANOVA followed by Dunnett's multiple comparisons test. ****P < 0.0001, ***P < 0.001, **P < 0.01.
